# Are Thyroid Autoimmune Diseases Associated with Cardiometabolic Risks in a Population with Normal Thyroid-Stimulating Hormone?

**DOI:** 10.1155/2018/1856137

**Published:** 2018-10-10

**Authors:** Yi Chen, Chunfang Zhu, Yingchao Chen, Ningjian Wang, Qin Li, Bing Han, Li Zhao, Chi Chen, Hualing Zhai, Lijuan Zhang, Yingli Lu

**Affiliations:** ^1^Institute and Department of Endocrinology and Metabolism, Shanghai Ninth People's Hospital, Shanghai Jiaotong University School of Medicine, Shanghai, China; ^2^Department of Endocrinology, Songjiang Hospital Affiliated to the First People's Hospital of Shanghai Jiaotong University, Shanghai, China

## Abstract

**Background:**

The interrelation between thyroid autoimmunity and cardiovascular risks is complex and has not been confirmed. This study aimed at evaluating whether there exists a relationship between thyroid autoimmune diseases (AITDs) and cardiometabolic risks in a large population with normal thyroid-stimulating hormone (TSH) levels.

**Methods:**

The data was obtained from a cross-sectional study (SPECT-China study). This study enrolled 9082 subjects (3948 males and 5134 females) above 18 years with normal TSH levels. AITD was defined according to the positivity of TPOAb and TgAb as well as thyroid ultrasonography (US) findings.

**Results:**

After full adjustment, TPOAb and/or TgAb positivity (TPO/TgAb (+)) was significantly associated with higher BMI, waist circumference (WC), and HbA1c only in women (*P* = 0.004, 0.026 and 0.032, respectively), while both TPO/TgAb positivity and US positivity (TPO/TgAb (+) and US (+)) were positively associated with BMI and WC in both genders (*P* = 0.002 and 0.020 in men; *P* < 0.001and <0.001 in women). TPO/TgAb (+) and US (+) were positively associated with HOMA-IR in women (*P* = 0.021) as well. Binary logistic analysis showed that AITDs had increased risks of central obesity, hyperlipidemia, and metabolic syndrome only in women (all *P* < 0.05). Moreover, TPO/TgAb (+) and US (+) were associated with an increased risk of obesity for both genders (*P* = 0.014 in men and *P* = 0.006 in women).

**Conclusions:**

Thyroid autoimmunity was positively associated with HbA1c, HOMA-IR, obesity, central obesity, hyperlipidemia, and metabolic syndrome, especially in women. This highlighted that AITDs may be potential risk factors for cardiometabolic disorders even if one's TSH was within the reference range.

## 1. Introduction

Autoimmune thyroid diseases (AITDs) comprise the most common autoimmune disease in humans. The basic mechanisms in the development of thyroid autoimmunity may be due to a combined TPO- and Tg-specific cytotoxic immune response [1]. High levels of either TPOAb or TgAb serve as a clinical marker for the detection of AITD [1–3] and they can be found in up to 20% in the “normal” population [4]. AITDs characterized by progressive destruction of thyroid tissue that may raise the level of TSH gradually lead to subclinical hypothyroidism and hypothyroidism [2, 5].

Thyroid hormones play important roles in regulating various processes of lipid and glucose metabolism, blood pressure, and energy expenditure [6]. The high prevalence of thyroid dysfunction including overt and subclinical hypothyroidism in the population has considerable consequences for a number of health issues, including insulin resistance, metabolic syndrome, dyslipidemia, central adiposity, and obesity which were involved as contributing factors to cardiometabolic risk (CVR) [7–10]. Recently, research has reported that there exists a close relationship of mild thyroid-stimulating hormone (TSH) variations within the reference range with a worse lipid profile, higher BMI, higher blood pressure, and the presence of metabolic syndrome in the general population [11–13]; however, conflicting studies were also present [14, 15].

Currently, most of the studies concerning the thyroid tissue and CVR focused on thyroid hormones (i.e., TSH, FT_3_, and FT_4_) instead of the direct role of thyroid autoimmunity (i.e., TPOAb positivity and TgAb positivity). The inflammatory pathway has a central role in the pathogenesis of both CVR and AITD. We hypothesize that there may exist common pathways between these two diseases. The association between thyroid autoimmunity and CVR is complex and has not been confirmed. The data used in this study was from a large investigation, the Survey on Prevalence in East China for Metabolic Diseases and Risk Factors (SPECT-China), which was performed in 2014-2015. The purpose of this study was to determine whether the prevalence of AITD was independently associated with CVR in a large Chinese population with normal TSH.

## 2. Materials and Methods

### 2.1. Study Participants

SPECT-China [16] is a population-based survey on the prevalence of metabolic diseases and risk factors in East China with a registration number of ChiCTR-ECS-14005052 (http://www.chictr.org.cn). A stratified and cluster-sampling method was used. From February 2014 to December 2015 [17, 18], this study was performed in Shanghai, Zhejiang, Jiangxi, Jiangsu, and Anhui Provinces, for a total of 22 sites in East China. Adults aged 18 years or older who were Chinese citizens and had lived in their current residence for more than 6 months were invited to participate in our study. Those with severe communication problems or severe/acute illness or who were unwilling to participate were excluded. All participants provided written informed consent before data collection. The study protocol was approved by the Ethics Committee of Shanghai Ninth People's Hospital, Shanghai Jiaotong University School of Medicine. All procedures followed were in accordance with the ethical standards of the responsible committee on human experimentation (institutional and national) and with the Helsinki Declaration of 1975, as revised in 2008 [17, 18].

**Figure 1:**
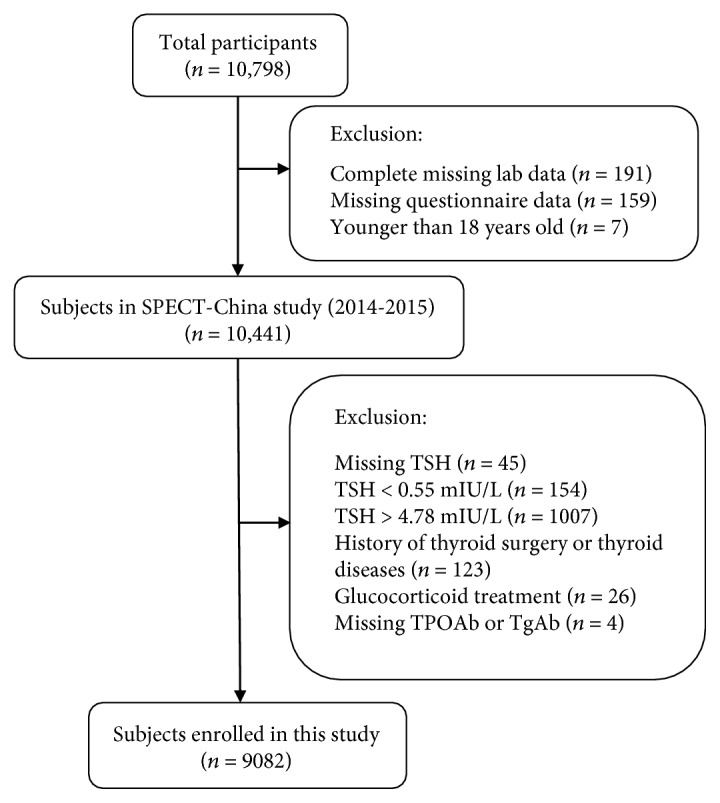
Flowchart of participants' inclusion and exclusion.

We followed the methods of Chen et al. in 2017 [19]. Our study initially enrolled 10,441 participants above 18 years old [17, 18]. Participants with missing TSH (*n* = 45), abnormal TSH levels (TSH < 0.55 mIU/L (*n* = 154), TSH > 4.78 mIU/L (*n* = 1007)), had a history that included thyroid surgery or thyroid diseases (including hyperthyroidism, hypothyroidism, subacute thyroiditis, and radioactive iodine treatment history) (*n* = 123), glucocorticoid treatment (*n* = 26), or missing TPOAb or TgAb levels (*n* = 4) were excluded. Finally, 9082 subjects were included in the final analysis. The inclusion and exclusion of participants in this analysis is shown in Figure 1.

### 2.2. Data Collection

At every step of this study, all data collection was performed by the same staff from the Department of Endocrinology and Metabolism in Shanghai Ninth People's Hospital, Shanghai Jiaotong University School of Medicine. All staff successfully completed a standard training program that made them familiar with the specific tools and methods used. A standard questionnaire was administered by trained staff to obtain information on demographic characteristics, personal and family medical history, and risk factors in their daily lives. Cardiovascular events including coronary heart disease, myocardial infarct, and stroke were recorded. Weight, height, and waist and hip circumference were measured according to a standard protocol. Blood pressure (BP) was measured at the nondominant arm 3 times consecutively with a 1-minute interval between the measurements with the participant in a seated position after 5 minutes of rest [20]. All anthropometric measurements were conducted at the same time when the serum samples were collected.

### 2.3. Laboratorial Assays

Serum samples for laboratorial assays were obtained by venipuncture after an 8-hour fast from 0700 to 1000 h in the morning. Blood samples were stored at −20°C when collected and shipped by air in dry ice to one central laboratory, which was certified by the College of American Pathologists (CAP), within 2–4 hours of collection.

TPOAb, TgAb, TSH, triiodothyronine (T_3_), and thyroxine (T_4_) were measured by the chemiluminescence immunoassay (Siemens, IMMULITE 2000, Erlangen, Germany). Fasting plasma glucose (FPG), low-density lipoprotein (LDL), high-density lipoprotein (HDL), triglycerides (TG), and total cholesterol (TC) were measured by Beckman Coulter AU680 (Brea, USA). Insulin was detected by chemiluminescence method (Abbott ARCHITECT i2000SR, Chicago, USA). Glycated hemoglobin (HbA1c) was assessed by high-performance liquid chromatography (MQ-2000PT, Medconn, Shanghai, China).

### 2.4. Thyroid Ultrasonography

Thyroid ultrasound examination was performed by the same registered physicians, who had a professional certificate for ultrasonography awarded by the Ministry of Health of China, using B-mode US imaging (M7 Premium, Shenzhen Mindray Bio-Medical Electronics Co. Ltd, P.R. China).

### 2.5. Definition of Variables

The normal reference range for TSH is 0.55–4.78 mIU/L; for TPOAb, it is 0–60 IU/mL; and for TgAb, it is 0–60 IU/mL.

AITD was defined as the serum TPOAb and/or TgAb positivity (>60 IU/mL) (TPO/TgAb (+)) and TPOAb and/or TgAb positivity together with characteristic ultrasonographic features (diffuse parenchymal hypoechogenicity and/or heterogeneous echogenic pattern of the thyroid gland) (TPO/TgAb (+) and US (+)) [18, 21, 22] for sensitive analysis.

Central obesity was defined as a waist circumference ≥ 80 cm in females and ≥90 cm in males [20]. Obesity was defined based upon BMI measures ≥ 30 kg/m^2^ [20]. Based on the American Diabetes Association 2014 criteria, diabetes was defined as a previous diagnosis by healthcare professionals, fasting plasma glucose ≥ 7.0 mmol/L, or HbA1c ≥ 6.5%. Hyperlipidemia was defined as total cholesterol ≥ 6.22 mmol/L, triglycerides ≥ 2.26 mmol/L, LDL-C ≥ 4.14 mmol/L or HDL-C < 1.04 mmol/L, or a self-reported previous diagnosis of hyperlipidemia by physicians. Hypertension was defined as a systolic blood pressure of 140 mmHg or higher or a diastolic blood pressure of 90 mmHg or higher or current use of antihypertensive treatment. Metabolic syndrome (MS) was defined based on the International Diabetes Federation criteria (2005). A person with MS must have abdominal obesity (waist circumference: male ≥ 90 cm, female ≥80 cm, or BMI ≥ 30 kg/m^2^) plus any two of the following four parameters: (1) raised TG ≥ 1.7 mmol/L, or treatment for this dyslipidemia; (2) reduced HDL < 1.03 mmol/L in men or HDL < 1.29 mmol/L in women, or treatment for this dyslipidemia; (3) raised blood pressure: systolic blood pressure ≥ 130 or diastolic blood pressure ≥ 85 mmHg, or treatment of hypertension; and (4) raised fasting plasma glucose ≥ 5.6 mmol/L or a history of type 2 diabetes [23].

### 2.6. Statistical Analysis

We performed survey analyses with IBM SPSS Statistics, Version 22 (IBM Corporation, Armonk, NY, USA). All analyses were two sided. A *P* value < 0.05 was considered significant. Continuous variables were expressed as the mean (±standard deviation) values, and categorical variables were presented as numbers (percentage). Continuous variables were compared using Student's *t*-test. The Mann–Whitney *U* test was used for nonnormally distributed continuous variables, and the Pearson *χ*^2^ test was used for dichotomous variables. The body mass index (BMI) was calculated as weight in kilograms divided by height in meters squared. Insulin resistance was estimated by the homeostatic model assessment (HOMA-IR) index: [fasting insulin (mIU/L)] × [FPG (mmol/L)]/22.5.

Associations among AITD and cardiometabolic risk factors were analyzed using linear regression models with each measure as the outcome. The regression models were adjusted for age, smoking history (including current and past), TSH, BMI (but not including obesity, central obesity, or metabolic syndrome in the regression model), and menopause status (age 50 used as a cutoff for menopause). TG, HbA1c, FPG, and HOMA-IR were ln transformed because of their skewed distribution. The results were expressed as *B* value and 95% confidence intervals (CIs).

The associations among AITD and cardiometabolic diseases (categorical variables) were assessed by logistic regression. The regression models were adjusted for age, smoking history (including current and past), TSH, BMI (but not included for obesity, central obesity or metabolic syndrome in the regression model), and menopause status (age 50 used as a cutoff for menopause). Results were expressed as odds ratios (95% confidence interval).

Sensitivity analyses were performed for the TPO/TgAb (+) and US (+) group.

## 3. Results

### 3.1. Clinical Characteristics according to TPOAb and TgAb Levels

A total of 9082 subjects (3948 males; 5134 females) with normal TSH levels were enrolled in this study. The mean age was 53.19 ± 13.13 years, and the mean body mass index (BMI) was 24.48 ± 3.51 kg/m^2^. The prevalence of TPOAb and/or TgAb positive (TPO/TgAb (+)) was 16.0% (10.1% in men and 20.6% in women). The characteristics of the study subjects in terms of TPO/TgAb positivity are summarized in Table 1. For men, participants with AITD had a significantly higher level of TSH and T_4_, a higher prevalence of subjects with cardiovascular events, and lower percentages of subjects with smoking history compared with participants in the TPOAb and TgAb negative (TPO and TgAb (−)) group. For women, participants with AITD had significantly higher BMI and waist circumference (WC), higher levels of TSH, T_4_, HOMA-IR, HbA1c, and triglycerides (TG), lower percentages of subjects with smoking history, and a higher prevalence of hyperlipidemia and metabolic syndrome.

### 3.2. Association of TPO/TgAb Positivity with Cardiometabolic Risk Factors

Table 2 summarizes the results of the linear regression models studying the association of TPO/TgAb positivity with cardiometabolic risk factors. TG, HbA1c, FPG, and HOMA-IR were all ln transformed because of their skewed distribution. After full adjustment for age, smoking history (including current and past), TSH, BMI (not included for BMI and waist circumference in regression model), and menopause status, TPO/TgAb positivity was significantly associated with BMI (*B* 0.353, 95% CI 0.112, 0.595), WC (*B* 0.712, 95% CI 0.086, 1.339), and HbA1c (*B* 0.010, 95% CI 0.001, 0.019) only in female participants, although no significant differences in this regard were noted in male subjects. Moreover, there was no significant association of TPO/TgAb positivity with HDL, LDL, TC, TG, FPG, HOMA-IR, and systolic blood pressure in both genders (*P* > 0.05).

### 3.3. Association of TPO/TgAb Positivity with Cardiometabolic Diseases

**Figure 2:**
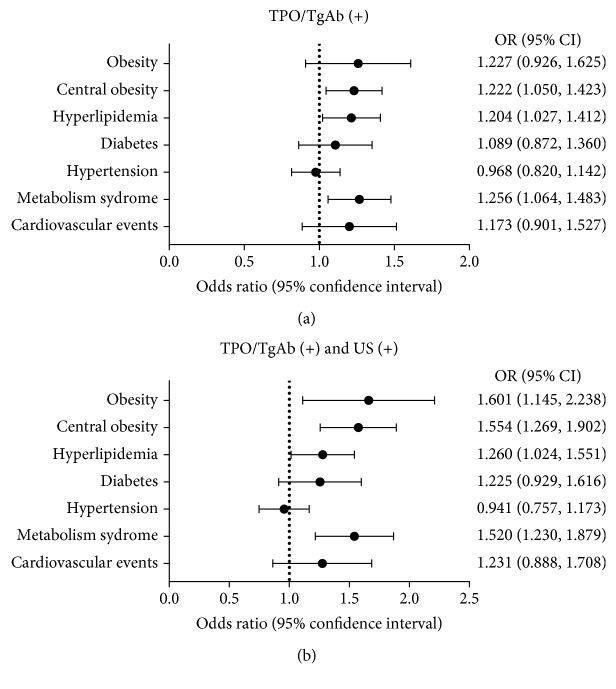
Associations of AITD with cardiometabolic diseases in women. They were analyzed using logistic regression. The regression models were adjusted for age, smoking history (including current and past), TSH, BMI (but not included for obesity or central obesity in the regression model), and menopause status. (a) TPO/TgAb (+) group; (b) TPO/TgAb (+) and US (+) group.

Given that the findings of TPO/TgAb positivity were positively associated with cardiometabolic risk factors, we evaluated the adjusted odds ratios (ORs) for cardiometabolic disease in the TPO/TgAb (+) group. Adjusted ORs were calculated after adjusting for age, smoking history (including current and past), TSH, BMI (but not included for obesity, central obesity, or metabolic syndrome in the regression model), and menopause status using the binary logistic regression model. As shown in Figure 2(a), TPO/TgAb positivity was associated with an increased risk of central obesity, hyperlipidemia, and metabolic syndrome in women. The ORs were 1.222 (95% CI 1.050, 1.423, *P* = 0.010), 1.204 (95% CI 1.027, 1.412, *P* = 0.022), and 1.256 (95% CI 1.064, 1.483, *P* = 0.007), respectively. Obesity, diabetes, hypertension, and cardiovascular events had no significant relationship with TPO/TgAb positivity. No significant association of TPO/TgAb positivity with cardiometabolic diseases were found in male subjects.

### 3.4. Sensitivity Analyses

After excluding 269 participants missing thyroid US information, 8813 participants (3811 males and 5002 females) were assessed for sensitivity analyses. The prevalence of TPOAb and/or TgAb positive together with characteristic US features (TPO/TgAb (+) and US (+)) was 7.4% (3.5% in men and 10.34% in women). The characteristics of the study subjects in terms of TPO/TgAb positivity and US positivity are summarized in Table 3.

TPO/TgAb positivity and US positivity were significantly associated with BMI in both genders (male: *B* 0.918, 95% CI 0.331, 1.504; female: *B* 0.779 95% CI 0.456, 1.102) and WC (male: *B* 1.998, 95% CI 0.321, 3.674; female: *B* 2.100 95% CI 1.263, 2.936). For women, TPO/TgAb positivity and US positivity were also significantly associated with HOMA-IR (*B* 0.071, 95% CI 0.010, 0.132). No other cardiometabolic risk factors were significantly associated with TPO/TgAb positivity and US positivity. (Table 2).

In terms of the relationship between TPO/TgAb (+) and US (+) and cardiometabolic diseases, the result was similar to the TPO/TgAb (+) group. Women with TPO/TgAb positivity and US positivity had a greater risk of central obesity, hyperlipidemia, and metabolic syndrome. The ORs were 1.554 (95% CI 1.269, 1.902, *P* < 0.001), 1.260 (95% CI 1.024, 1.551, *P* = 0.029), and 1.520 (95% CI 1.230, 1.879, *P* < 0.001), respectively (Figure 2(b)). Moreover, TPO/TgAb positivity and US positivity also had a significantly association with obesity in both genders (male: OR 1.982, 95% CI 1.149, 3.420, *P* = 0.014; female: OR 1.601, 95% CI 1.145, 2.238, *P* = 0.006).

## 4. Discussion

Our present evaluation of the association of thyroid autoimmunity with cardiometabolic diseases and risk factors according to the TPOAb and (or) TgAb positivity and US findings has revealed that thyroid autoimmunity played an independent role in cardiometabolic disorders, especially in women.

The Tehran Lipid and Glucose Study [24] showed that the frequency of positive TPOAb in never smokers was significantly higher than ever smokers. Similarly, our data showed that the frequency of smoke was decreased in participants with AITD. A significant difference was detected in women. Smoking has significant effects on thyroid function and could be a potential confounder to assess the association between AITD and metabolic syndrome [24, 25]. In this study, all analyses were performed with adjustment for smoking.

Only scanty studies about the association of thyroid autoimmunity with obesity and central obesity in a large Chinese population with a normal TSH level were available. This study showed that thyroid autoimmunity was positively associated with BMI and WC in women. Obesity and central obesity were increased by about 1.60- and 1.55-fold in the presence of both TPO/TgAb positivity and US positivity. For men, the adjusted odds ratio of obesity increased by 98% in the TPO/TgAb (+) and US (+) group. Thus, our data suggested that thyroid autoimmunity had a close relationship with obesity and central obesity.

We also found in women that both TPO/TgAb (+) patients and TPO/TgAb (+) together with US (+) patients had higher serum TC, TG, and LDL levels and lower HDL levels, but only TG had a statistically significant change. Besides these lipid profiles, previous diagnosis of hyperlipidemia was also considered in this study. After full adjustment, patients with AITD had more risk for hyperlipidemia. Some other studies reached a similar conclusion that positive TPOAb can cause dyslipidemia in the euthyroid population [26, 27], while Wells and Hueston recorded no significant difference between the TPOAb-positive group and TPOAb-negative group [28].

In terms of glucose metabolism, several studies have suggested a major role for autoimmunity in the pathogenesis of type 1 diabetes mellitus (T_1_DM). Thyroid antibodies were frequent in patients with T_1_DM [29, 30]. A case-control study [31] involving three hundred and two type 2 diabetes mellitus (T_2_DM) patients and three hundred and ten nondiabetic controls showed that TPOAb level was significantly higher among T_2_DM subjects compared to controls, and thyroid autoimmunity in T_2_DM subjects was significantly associated with poor glycemic control. In line with this study, our data showed in women that the TPO/TgAb (+) was positively associated with HbA1c and TPO/TgAb and US (+) was positively associated with HOMA-IR.

Metabolic syndrome is an important predictor of cardiometabolic risk and is a cluster of abnormalities such as obesity (including central obesity), dyslipidemia, high plasma glucose, and hypertension [32]. The findings of female gender dominance in the prevalence of metabolic syndrome were seen in several studies [33, 34] which showed the similar tendency with the prevalence of AITD [35]. In addition, higher TSH and IL-6 concentrations were detected in metabolic syndrome [36] while AITD was reported to be associated with higher TSH and IL-6 levels as well [1, 5]. We therefore hypothesized that there may be a crosstalk between AITD and metabolic syndrome. Our present findings suggested that women with AITD had a greater risk of metabolic syndrome. The adjusted odds ratio of metabolic syndrome was increased by about 25.6% and 52.0% in the TPO/TgAb (+) group and TPO/TgAb (+) and US (+) group. However, conflicting evidence has been reported. A study including three hundred and seventy-two postmenopausal women showed that the prevalence of metabolic syndrome was similar between the TPOAb-positive group and the TPOAb-negative group in euthyroid women. Obese subclinical hypothyroid women with Hashimoto's thyroditis have a higher prevalence of metabolic syndrome [36]. Agbaht et al. pointed out from their study including five hundred and eighty-four participants that TPOAb level had no association with metabolic syndrome, even in the obese population [37]. In terms of different ages, race, and ethnicity, gender composition may have accounted for the difference in results obtained. Additionally, compared with the above studies, our survey with such a large sample size maybe more reliable.

Our results confirmed a close relationship between AITD and cardiometabolic disorders, especially in women. In general, autoimmune diseases including AITD are much more common in females [38], and the immune response is greater in females than in males as well [39]. It is well established that the immune system exhibits sexual dimorphism in humans. That is, AITD affects males and females differently [40, 41].

Our study has several strengths. First, we evaluated a relatively large sample of participants to examine the association of TPOAb and TgAb with cardiometabolic risks. Second, the data were highly reliable because all the anthropometric measurements and questionnaires were completed by the same trained research group using strong quality control, and all the tests were performed with the same equipment and protocol. Third, compared to a clinic-based population, community-dwelling participants living in multiple sites in China were recruited so that the results may be more representative.

However, several limitations of this study should also be considered. First, because of the cross-sectional study, the direction of any causal relationship could not be established. Prospective studies are needed. Second, we used an age proxy to define postmenopausal status. In China, the overall median age at natural menopause is 50 years [42]. Third, no association between AITDs and cardiovascular events was found in this study. However, it must be noted that those with severe communication problems or severe/acute illness (maybe a result from these cardiovascular events) were excluded in this study, which may have introduced bias. Fourth, we did not measure levels of FT_3_ and FT_4_ and thus could not assess their probable role in metabolic syndrome parameters.

## 5. Conclusion

Thyroid autoimmunity was positively associated with HbA1c, HOMA-IR, central obesity, hyperlipidemia, and metabolic syndrome, especially in women. It was also positively associated with obesity in both genders. This highlighted that AITD may be a potential independent risk factor for cardiometabolic disorders even if one's TSH was among the reference range.

## Figures and Tables

**Table 1:** Characteristics of subjects in terms of the level of serum TPOAb and TgAb.

	Total	TPO and TgAb (−)	TPO/TgAb (+)	*P* value
*Men*				
*N* (%)	3948 (100)	3548 (89.9)	400 (10.1)	—
Age (year)	53.93 ± 13.12	53.91 ± 13.05	54.13 ± 13.82	0.753
Smokers (%)	55.3	55.8	51.3	0.095
Systolic BP (mmHg)	134.31 ± 20.66	134.41 ± 20.80	133.42 ± 19.45	0.347
BMI (kg/m^2^)	24.82 ± 3.35	24.80 ± 3.35	25.04 ± 3.36	0.174
WC (cm)	84.23 ± 9.60	84.22 ± 9.60	84.37 ± 9.55	0.777
FPG (mmol/L)	5.70 ± 1.52	5.72 ± 1.56	5.55 ± 1.15	0.481
HOMA-IR	1.47 ± 2.21	1.48 ± 2.30	1.32 ± 1.15	0.461
HbA1c (%)	5.61 ± 1.01	5.62 ± 1.02	5.52 ± 0.91	0.112
LDL (mmol/L)	3.07 ± 0.76	3.07 ± 0.77	3.06 ± 0.69	0.807
HDL (mmol/L)	1.36 ± 0.32	1.36 ± 0.32	1.35 ± 0.30	0.557
TG (mmol/L)	1.88 ± 1.95	1.90 ± 2.02	1.74 ± 1.19	0.856
TC (mmol/L)	5.15 ± 1.13	5.16 ± 1.14	5.11 ± 1.00	0.400
TSH (mIU/L)	2.13 ± 0.92	2.11 ± 0.91	2.36 ± 0.99	<0.001
T_3_ (nmol/L)	1.79 ± 0.53	1.77 ± 0.42	1.94 ± 1.10	0.152
T_4_ (nmol/L)	113.87 ± 22.34	113.64 ± 22.42	116.00 ± 21.43	0.047
Hyperlipidemia (%)	42.2	42.2	42.3	0.972
Diabetes (%)	14.9	15.1	13.5	0.392
Hypertension (%)	51.3	51.7	48.2	0.193
Metabolic syndrome (%)	20.3	20.4	19.3	0.604
Cardiovascular events (%)	7.4	7.1	10.3	0.021
*Women*				
*N* (%)	5134 (100)	4077 (79.4)	1057 (20.6)	—
Age (year)	52.62 ± 13.11	52.56 ± 13.20	52.83 ± 12.77	0.562
Smokers (%)	2.8	3.0	1.8	0.036
Systolic BP (mmHg)	130.19 ± 22.28	129.99 ± 22.06	130.97 ± 23.11	0.207
BMI (kg/m^2^)	24.21 ± 3.60	24.13 ± 3.56	24.54 ± 3.74	0.002
WC (cm)	77.71 ± 9.99	77.54 ± 10.00	78.38 ± 9.92	0.017
FPG (mmol/L)	5.56 ± 1.34	5.56 ± 1.32	5.57 ± 1.42	0.623
HOMA-IR	1.56 ± 1.95	1.53 ± 1.77	1.67 ± 2.54	0.017
HbA1c (%)	5.45 ± 0.90	5.43 ± 0.88	5.52 ± 0.96	0.001
LDL (mmol/L)	3.06 ± 0.79	3.05 ± 0.79	3.10 ± 0.81	0.096
HDL (mmol/L)	1.48 ± 0.31	1.48 ± 0.32	1.47 ± 0.31	0.313
TG (mmol/L)	1.48 ± 1.14	1.46 ± 1.04	1.58 ± 1.47	0.003
TC (mmol/L)	5.14 ± 1.13	5.15 ± 1.16	5.15 ± 1.03	0.994
TSH (mIU/L)	2.46 ± 1.00	2.42 ± 1.00	2.59 ± 1.02	<0.001
T_3_ (nmol/L)	1.73 ± 0.40	1.72 ± 0.36	1.77 ± 0.50	0.018
T_4_ (nmol/L)	115.49 ± 19.59	115.17 ± 19.20	116.73 ± 20.99	0.029
Hyperlipidemia (%)	31.3	30.4	34.7	0.006
Diabetes (%)	12.0	11.7	13.1	0.230
Hypertension (%)	41.1	40.8	42.1	0.449
Metabolic syndrome (%)	26.5	25.6	29.8	0.007
Cardiovascular events (%)	7.5	7.3	8.5	0.168

*P* value: TPO/TgAb (+) versus TPO and TgAb (−). Data are presented as the mean ± standard deviation for continuous variables or as a percentage (%) for categorical variables. BP, blood pressure; BMI, body mass index; WC, waist circumference; FPG, fasting blood glucose; HOMA-IR, homeostasis model assessment of insulin resistance; HbA1c, glycated hemoglobin; LDL, low-density lipoprotein; HDL, high-density lipoprotein; TG, triglycerides; TC, total cholesterol; TSH, thyroid-stimulating hormone; T_3_, triiodothyronine; T_4_, thyroxin.

**Table 2:** Associations of AITD with cardiometabolic risk factors by linear regression.

	Men				Women			
	TPO/TgAb (+)		TPO/TgAb and US (+)		TPO/TgAb (+)		TPO/TgAb and US (+)	
	*B* (95% CI)	*P*	*B* (95% CI)	*P*	*B* (95% CI)	*P*	*B* (95% CI)	*P*
BMI	0.171 (−0.186, 0.527)	0.349	0.918 (0.331, 1.504)	0.002	0.353 (0.112, 0.595)	0.004	0.779 (0.456, 1.102)	<0.001
WC	0.097 (−0.925, 1.120)	0.852	1.998 (0.321, 3.674)	0.020	0.712 (0.086, 1.339)	0.026	2.100 (1.263, 2.936)	<0.001
HDL	−0.004 (−0.037, 0.028)	0.792	0.011 (−0.042, 0.064)	0.691	0.001 (−0.021, 0.022)	0.950	−0.004 (−0.033, 0.024)	0.778
LDL	−0.004 (−0.046, 0.006)	0.924	0.021 (−0.107, 0.149)	0.749	0.026 (−0.026, 0.078)	0.331	−0.011 (−0.081, 0.059)	0.751
TC	−0.030 (−0.150, 0.090)	0.625	0.066 (−0.130, 0.262)	0.508	−0.017 (−0.092, 0.059)	0.662	−0.020 (−0.121, 0.081)	0.700
TG	−0.033 (−0.092, 0.027)	0.281	−0.021 (−0.119,0.076)	0.667	0.032 (0.000, 0.065)	0.052	0.035 (−0.009, 0.078)	0.121
HbA1c	−0.014 (−0.030, 0.002)	0.093	−0.004 (−0.030, 0.023)	0.785	0.010 (0.001, 0.019)	0.032	0.011(−0.002, 0.023)	0.089
FPG	−0.021 (−0.042, 0.000)	0.055	0.006 (−0.028, 0.041)	0.724	−0.005 (−0.017, 0.007)	0.444	0.003 (−0.013, 0.019)	0.718
HOMA-IR	−0.011 (−0.083, 0.062)	0.771	0.058 (−0.061, 0.177)	0.340	0.032 (−0.013, 0.077)	0.159	0.071 (0.010, 0.132)	0.021
Systolic BP	−1.509 (−3.536, 0.519)	0.145	−1.071 (−4.418, 2.276)	0.531	0.173 (−1.159, 1.505)	0.799	−0.309 (−2.091, 1.474)	0.731

TG, HbA_1_c, FPG, and HOMA-IR was ln transformed for normal distribution. The regression models were adjusted for age, smoking history (including current and past), TSH, BMI (not included for BMI and WC in regression model), and menopause status in women.

**Table 3:** Characteristic of subjects in terms of TPOAb, TgAb, and US.

	TPO and TgAb (−)	TPO/TgAb (+) and US (+)	*P* value
*Men*			
*N* (%)	3426 (89.9)	134 (3.5)	—
Age (year)	53.80 ± 13.04	54.52 ± 13.09	0.527
Smokers (%)	55.6	49.6	0.177
Systolic BP (mmHg)	134.36 ± 20.81	135.56 ± 20.68	0.516
BMI (kg/m^2^)	24.83 ± 3.35	25.75 ± 3.45	0.002
WC (cm)	84.26 ± 9.60	86.21 ± 9.29	0.022
FPG (mmol/L)	5.72 ± 1.56	5.22 ± 1.18	0.185
HOMA-IR	1.49 ± 2.32	1.43 ± 0.98	0.038
HbA1c (%)	5.62 ± 1.02	5.61 ± 0.89	0.721
LDL (mmol/L)	3.07 ± 0.76	3.14 ± 0.68	0.351
HDL (mmol/L)	1.36 ± 0.32	1.34 ± 0.31	0.543
TG (mmol/L)	1.91 ± 1.99	1.84 ± 1.22	0.187
TC (mmol/L)	5.16 ± 1.13	5.22 ± 1.18	0.536
TSH (mIU/L)	2.11 ± 0.91	2.56 ± 1.02	<0.001
T_3_ (nmol/L)	1.77 ± 0.42	1.73 ± 0.36	0.127
T_4_ (nmol/L)	113.59 ± 22.30	110.13 ± 19.24	0.077
Hyperlipidemia (%)	42.7	49.3	0.130
Diabetes (%)	15.0	17.9	0.363
Hypertension (%)	51.6	50.8	0.852
Metabolic syndrome (%)	20.5	24.6	0.257
Cardiovascular events (%)	7.2	9.0	0.429
*Women*			
*N* (%)	3970 (79.4)	517 (10.3)	—
Age (year)	52.45 ± 13.16	54.56 ± 12.31	<0.001
Smokers (%)	2.9	1.4	0.053
Systolic BP (mmHg)	129.93 ± 22.07	132.72 ± 23.65	0.008
BMI (kg/m^2^)	24.16 ± 3.56	25.11 ± 3.73	0.002
WC (cm)	77.53 ± 10.00	80.32 ± 10.05	<0.001
FPG (mmol/L)	5.55 ± 1.30	5.67 ± 1.53	0.061
HOMA-IR	1.53 ± 1.77	1.78 ± 2.86	<0.001
HbA1c (%)	5.43 ± 0.87	5.59 ± 0.99	<0.001
LDL (mmol/L)	3.05 ± 0.79	3.11 ± 0.81	0.084
HDL (mmol/L)	1.48 ± 0.32	1.46 ± 0.31	0.088
TG (mmol/L)	1.46 ± 1.04	1.71 ± 1.88	<0.001
TC (mmol/L)	5.14 ± 1.16	5.20 ± 1.03	0.280
TSH (mIU/L)	2.43 ± 0.99	2.68 ± 1.05	<0.001
T_3_ (nmol/L)	1.72 ± 0.36	1.75 ± 0.53	0.644
T_4_ (nmol/L)	115.10 ± 19.16	115.31 ± 21.45	0.833
Hyperlipidemia (%)	30.4	38.9	<0.001
Diabetes (%)	11.5	15.7	0.006
Hypertension (%)	40.6	46.4	0.013
Metabolic syndrome (%)	25.5	35.8	<0.001
Cardiovascular events (%)	7.4	10.1	0.033

Data are presented as the mean ± standard deviation for continuous variables or as a percentage (%) for categorical variables. BP, blood pressure; BMI, body mass index; WC, waist circumference; FPG, fasting blood glucose; HOMA-IR, homeostasis model assessment of insulin resistance; HbA1c, glycated hemoglobin; LDL, low-density lipoprotein; HDL, high-density lipoprotein; TG, triglycerides; TC, total cholesterol; TSH, thyroid-stimulating hormone; T_3_, triiodothyronine; T_4_, thyroxin.

## Data Availability

The data used to support the findings of this study are available from the corresponding author upon request.

## Abbreviations

TPOAb: Thyroid peroxidase antibody
TgAb: Thyroglobulin antibody
US: Ultrasonography
AITDs: Thyroid autoimmune diseases
CVR: Cardiometabolic risk
BP: Blood pressure
BMI: Body mass index
WC: Waist circumference
FPG: Fasting blood glucose
HOMA-IR: Homeostasis model assessment of insulin resistance
HbA1c: Glycated hemoglobin
LDL: Low-density lipoprotein
HDL: High-density lipoprotein
TG: Triglycerides
TC: Total cholesterol
TSH: Thyroid-stimulating hormone
T_3_: Triiodothyronine
T_4_: Thyroxine.
